# Cause of death in Washington state veterans hospitalized with acute coronary syndromes in the veterans health administration

**DOI:** 10.1186/1478-7954-6-3

**Published:** 2008-07-23

**Authors:** Charles Maynard, Elliott Lowy, Mary McDonell, Stephan D Fihn

**Affiliations:** 1 Department of Veterans Affairs Puget Sound Health Care System, Seattle, WA, USA; 2 Department of Health Services, University of Washington, Seattle, WA, USA; 3 Department of Medicine, University of Washington, Seattle, WA, USA

## Abstract

**Background:**

In the United States, relatively little is known about cause of death in individuals who die prior to or after hospital discharge for acute coronary syndromes (ACS). The purpose of this report was to compare baseline patient characteristics according to whether the underlying cause of death was cardiac or non-cardiac.

**Methods:**

We linked cause of death information from Washington State death records to the Department of Veterans Affairs (VA) External Peer Review Program ACS registry. From 524 individuals who were hospitalized for ACS in veterans hospitals located in Washington State or Oregon, we identified 136 individuals who according to VA death records died during the years 2003 to 2005. Of these, 117 (86%) were found in Washington State death records. Sociodemographic variables, as well as underlying and secondary causes of death, were obtained from Washington State death records provided by the Washington State Department of Health. Clinical variables, including medical histories, presentation on admission, and in-hospital death were extracted from the VA ACS registry.

**Results:**

Somewhat surprisingly, only 52% of veterans died of cardiac causes when only the underlying cause of death was used. However, when secondary causes of death were added to the definition, the proportion that died of cardiac causes increased to 81%. Patient characteristics were similar in the two groups, although small numbers limited the ability to detect statistically significant differences.

**Conclusion:**

These preliminary findings suggest that it is important to consider secondary causes as well as the underlying one when classifying deaths as cardiac or non-cardiac.

## Background

In the United States, relatively little is known about cause of death in individuals who die prior to or after hospital discharge for acute coronary syndromes (ACS). Many large registries such as the National Registry of Myocardial Infarction do not collect information on patients after hospitalization for ACS, nor do they collect information regarding the circumstances of death occurring in the hospital. The situation in Europe is somewhat different in that it is possible to link national vital status registers with disease based registries [1,2]. The National Death Index in the United States is a means for ascertaining vital status and cause of death, but search charges can be expensive for disease registries with large numbers of patients [3]. The Department of Veterans Affairs (VA) devotes considerable resources to chart abstraction for patients hospitalized with ACS and also has a vital status register that does not contain cause of death information [4,5]. The major objective of this report was to compare baseline patient characteristics according to whether the underlying cause of death was cardiac or non-cardiac. This was accomplished by linking cause of death information from Washington State death records to the VA External Peer Review Program ACS registry.

## Methods

### Patient population

Between October 2003 and December 2005, there were 524 individuals who were admitted to VA hospitals located in Washington State or Oregon and had a diagnosis of acute myocardial infarction or unstable angina. These individuals were Washington State residents (identified by residential zip code) admitted to VA hospitals or were non-state residents admitted to 1 of 3 VA hospitals in Washington State. According to VA death records, 136 (26%) died prior to January 1, 2006. Using the social security number, full patient name, and date of birth, these records were linked to death records obtained from the Washington State Department of Health. This process identified 117 (86%) decedents in state death records. Of the 19 who were not found, 8 were Washington State residents, 6 were residents of Idaho, 3 – of Alaska, 1 – of Montana, and 1 – of Oregon. Individuals with missing death records were similar to those whose death records were found in that the two groups did not differ with respect to age, gender, race, or year of death.

### Study variables

Sociodemographic variables in this study were obtained from Washington State death records and included age, gender, race, education, marital status, tobacco use as contributing to death, underlying and secondary causes of death, for which there were up to 20 codes. Cause of death was categorized as cardiac or non-cardiac, and International Classification of Diseases 10^th ^Revision (ICD-10) codes were used to classify cause of death. The following ranges of ICD-10 codes were used to identify cardiac causes: I10 – I15 (hypertensive disease), I20 – I25 (ischemic heart disease), and I30 – I52 (other forms of heart disease). Cause of death classified as cardiac or non-cardiac was defined in two ways; first, only the underlying cause of death was considered, and second, both underlying and secondary ICD-10 codes were used. Variable collection for the External Peer Review Program database has been described in detail [4]. Using this database we determined the type of ACS (defined as ST elevation myocardial infarction, non ST elevation myocardial infarction, or unstable angina) and whether the patient died in the hospital. Numerous other patient characteristics were collected, including heart failure, prior coronary artery bypass graft surgery, lipid disorder, diabetes mellitus, tobacco use, dementia, and cerebrovascular, chronic obstructive pulmonary, and renal diseases. Cardiac medications prescribed at admission were also recorded. This study was approved as by the University of Washington Human Subjects Review Board. A waiver of informed consent was granted.

### Statistical methods

For comparing differences between the cardiac and non-cardiac groups, the 2 sample t-test was used for continuous variables and the Chi-square statistic – for categorical variables.

## Results

The distribution of underlying cause of death is presented in table 1; 52% of deaths were due to cardiac causes of which over 90% were due to ischemic conditions. Individuals in this study can be characterized as older white men, 32% of whom did not graduate from high school (Table 2). A higher proportion of the non-cardiac group included African-American veterans, but for the most part socio-demographic characteristics were similar in the two groups. Of the 7 African-Americans, 1 (14%) died of a cardiac cause, whereas over half of 100 white patients did. Clinical characteristics in the two groups were similar with the exception that a higher proportion of the non-cardiac group included individuals with a history of cerebrovascular disease (Table 3). Of the 11 individuals with a history of cerebrovascular disease, 2 (18%) died of cardiac causes, whereas 56% of those without a history of cerebrovascular disease died of cardiac causes.

**Table 1:** Distribution of underlying cause of death (n = 117)

Cause	n (%)
**Non-cardiac**	56 (48%)
Infection	4 (3%)
Cancer	18(15%)
Diabetes	4 (3%)
Dementia	1 (< 1%)
Polyneuropathy	1 (< 1%)
Stroke	2 (2%)
Vascular	2 (2%)
Pulmonary	10 (8%)
Gastric	4 (3%)
Decubitus ulcer	1 (< 1%)
Renal	7 (6%)
Accident	2 (2%)
**Cardiac**	61(52%)
Hypertension	1 (< 1%)
Acute myocardial infarction	23 (20%)
Atherosclerotic cardiovascular disease	32 (27%)
Other ischemic heart disease	2 (2%)
Atrial fibrillation	1 (< 1%)
Cardiomyopathy	1 (< 1%)
Congestive heart failure	1 (< 1%)

**Table 2:** Socio-demographic characteristics of decedents according to underlying cause of death

Characteristic	Cardiac (n = 61)	Non-cardiac (n = 56)	Total (n = 117)	P
Year of death				0.86
2003	7%	5%	6%	
2004	39%	36%	38%	
2005	54%	59%	56%	
Age (yrs)	74 ± 10	76 ± 9	75 ± 10	0.18
Men	97%	95%	96%	
Race				0.033
White	93%	89%	92%	
African-American	2%	11%	6%	
Other	5%	0%	2%	
Marital status				0.38
Never	13%	9%	11%	
Married	51%	43%	47%	
Widowed	23%	23%	23%	
Divorced	13%	25%	19%	
Education				0.07
< 12 years	21%	43%	32%	
High school graduate	46%	39%	43%	
Some college	21%	12%	17%	
College graduate	12%	5%	8%	
Did tobacco use contribute to death?				0.23
Yes	26%	14%	20%	
No	23%	36%	29%	
Probably	12%	7%	9%	
Unknown	11%	43%	41%	

**Table 3:** Clinical characteristics of decedents according to underlying cause of death

Characteristic	Cardiac (n = 61)	Non-cardiac (n = 56)	Total (n = 117)	P
History of				
Congestive heart failure	52%	48%	50%	0.26
Coronary artery bypass graft surgery	28%	16%	22%	0.12
Myocardial infarction	41%	30%	36%	0.23
Lipid disorder	57%	50%	54%	0.42
Cerebrovascular disease	3%	16%	9%	0.018
Chronic obstructive pulmonary disease	28%	41%	34%	0.13
Diabetes mellitus	23%	16%	20%	0.35
Renal disease	15%	18%	16%	0.65
Tobacco use	50%	39%	44%	0.44
Dementia	20%	29%	24%	0.26
Medications				
Angiotensin converting enzyme inhibitor	57%	45%	51%	0.17
Aspirin	57%	52%	55%	0.54
Beta-blockers	62%	46%	55%	0.08
Lipid lowering	62%	45%	54%	0.056
Platelet inhibitors	15%	20%	17%	0.48
Type of acute coronary syndrome				0.17
Unstable angina	7%	4%	5%	
Non ST elevation myocardial infarction	78%	91%	85%	
ST elevation myocardial infarction	15%	5%	10%	
In-hospital death	26%	30%	28%	0.62

When secondary ICD-10 codes were considered, the number of deaths due to cardiac causes increased by 39%, from 61 to 95, and the percent with death due to cardiac causes rose to 81%. In 34 individuals with only a secondary diagnosis of cardiac disease, the underlying causes of death were cancer (26%), respiratory (20%), genitourinary, including renal (12%), vascular, including cerebrovascular (12%), diabetes (9%), digestive (9%), and other (12%) including bacterial, mental, and accidental. For both demographic and clinical characteristics, the 2 groups were similar, although the level of education was higher in the cardiac group and there were proportionally more African-Americans in the non-cardiac group (Tables 4 and 5).

**Table 4:** Socio-demographic characteristics of decedents according to underlying and secondary causes of death

Characteristic	Cardiac (n = 95)	Non-cardiac (n = 22)	Total (n = 117)	P
Year of death				0.79
2003	5%	9%	6%	
2004	38%	36%	38%	
2005	57%	55%	56%	
Age (yrs)	74 ± 10	77 ± 10	75 ± 10	0.22
Men	96%	96%	96%	0.94
Race				0.18
White	93%	86%	92%	
African-American	4%	14%	6%	
Other	3%	0%	2%	
Marital status				0.23
Never	14%	0%	11%	
Married	47%	46%	47%	
Widowed	22%	27%	23%	
Divorced	17%	27%	19%	
Education				0.006
< 12 years	26%	54%	32%	
High school graduate	48%	18%	43%	
Some college	15%	27%	17%	
College graduate	11%	0%	8%	
Did tobacco use contribute to death?				0.49
Yes	22%	14%	20%	
No	26%	41%	29%	
Probably	10%	9%	9%	
Unknown	37%	27%	41%	

**Table 5:** Clinical characteristics of decedents according to underlying and secondary causes of death

Characteristic	Cardiac (n = 95)	Non-cardiac (n = 22)	Total (n = 117)	P
History of				
Congestive heart failure	50%	54%	50%	0.67
Coronary artery bypass graft surgery	24%	14%	22%	0.28
Myocardial infarction	38%	27%	36%	0.35
Lipid disorder	56%	46%	54%	0.38
Cerebrovascular disease	10%	9%	9%	0.96
Chronic obstructive pulmonary disease	35%	32%	34%	0.80
Diabetes mellitus	22%	9%	20%	0.17
Renal disease	16%	18%	16%	0.78
Tobacco use	44%	46%	44%	0.93
Dementia	22%	32%	24%	0.34
Medications				
Angiotensin converting enzyme inhibitor	52%	50%	51%	0.89
Aspirin	56%	50%	55%	0.62
Beta-blockers	57%	46%	55%	0.33
Lipid lowering	56%	46%	54%	0.38
Platelet inhibitors	17%	18%	17%	0.88
Type of acute coronary syndrome				0.27
Unstable angina	6%	0%	5%	
Non ST elevation myocardial infarction	82%	96%	85%	
ST elevation myocardial infarction	12%	4%	10%	
In-hospital death	30%	23%	28%	0.53

## Discussion

It was somewhat surprising that only about half of the individuals hospitalized for ACS died of cardiac causes when only the underlying cause of death was used to identify cardiac deaths. However, when secondary causes were included in the definition, over 80% of deaths were due to cardiac conditions. This result seems more reasonable given that decedents had significant cardiovascular disease and were hospitalized for either acute myocardial infarction or unstable angina pectoris. The findings that there were proportionally more African-Americans as well as lower levels of education in both non-cardiac groups are intriguing and need further investigation. There were, however, only seven African-Americans in the study. The ability to detect statistically significant differences was limited by small numbers of deaths.

This study also demonstrated that it was possible to link state death records with the VA ACS database, although we were unable to find Washington State death records for 19 individuals (8 Washington State residents, 11 non residents) who were deceased according to VA records. In theory, Washington State death records include all deceased residents, even those who died out of state, and may include non-state residents who died in the state. In practice, this may be quite different as states have varying reciprocal reporting arrangements. It is also possible that reporting of deaths was delayed, and that some of these missing deaths may appear in subsequent annual updates provided by the state, although this was not the case in 2006. We did not have the time or resources to contact neighboring states to determine the vital status of the 11 non-Washington State residents whose death records were not found.

In general, cause of death information, despite its inherent limitations, is potentially useful to researchers, managers, and policy makers [6]. There are long standing concerns about the accuracy of this information as well as about who determines the cause of death. However, a recent study reported that Washington State veterans who used VA health care were more likely to die of alcohol and/or drug related causes than veterans who did not use VA health care [7]. While such information may be of value, it can be difficult to obtain. The United States National Center for Health Statistics does support the National Death Index, which along with the Social Security Death Master File, are the major methods of mortality ascertainment in the United States [3]. The National Death Index, however, is the only national source of cause of death information in the United States and is available only for research purposes. Only a small number of states such as Washington make their death records available to researchers.

## Conclusion

In the future, we expect that more relevant findings will be generated as deaths accrue in Washington State veterans with ACS. We also hope to use the National Death Index for further investigation. These preliminary findings suggest that it is important to consider secondary causes as well as the underlying one when classifying deaths as cardiac or non-cardiac.

## Competing interests

The authors declare that they have no competing interests.

## Authors' contributions

CM assumed responsibility for study design, conception, data acquisition, analysis and interpretation, drafted the manuscript, critically revised it for important intellectual content, and approved the final version. EL, MM, and SDF were responsible for data acquisition, analysis and interpretation, critically revised the paper for important intellectual content, and approved the final version of manuscript.
